# An unusual pseudolymphoma in the context of necrotizing fasciitis: A case report

**DOI:** 10.1097/MD.0000000000032457

**Published:** 2022-12-23

**Authors:** Bastian Dislich, Dennis Hoch, Stefan Dirnhofer, Urban Novak, Yara Banz

**Affiliations:** a Institute of Pathology, University of Bern, Bern, Switzerland; b Department of Medical Oncology, Inselspital, Bern University Hospital, Bern, Switzerland; c Institute of Pathology, University Hospital Basel, Basel, Switzerland.

**Keywords:** blast, case report, infection, lymph node, plasmablast, pseudolymphoma

## Abstract

The diagnosis of lymphoma in routine diagnostics can be challenging due to clinical, morphological and immunphenotypical overlap with unusual reactive processes termed “pseudolymphomas.”

**Patient concerns:**

45-year-old male that underwent surgical debridement for a necrotizing fasciitis of the thigh with concomitant excision of a regional lymph node.

**Diagnoses:**

The lymph node demonstrated an architecture-effacing activation and proliferation of lymphoblasts and was initially misdiagnosed as an aggressive lymphoma. Only in consideration of the clinical context and with the help of additional immunohistochemical and molecular analyses the final diagnosis of a reactive lymphadenopathy could be made.

**Interventions:**

No further therapy was required after the final diagnosis of a reactive lymphadenopathy was made.

**Outcomes:**

The clinical follow-up was unremarkable, with no evidence of residual disease after 6 months.

**Lessons:**

This case report adds the parafollicular activation and proliferation of blasts and plasmablasts in the drainage area of an active infection to the spectrum of “pseudolymphomas” and reiterizes the importance of placing histopathological findings in the proper context.

## 1. Introduction

The diagnosis of most lymphomas relies on the combined clinical, morphological and immunophenotypical findings, with the addition of ancillary molecular studies in difficult cases or where the molecular aberration defines the specific entity. However, a significant clinical, morphological and immunphenotypical overlap exists between true neoplastic lesions and unusual reactive processes termed “pseudolymphomas.” This may result in the misdiagnosis of reactive processes as lymphomas. Awareness of the specific clinical context in which pseudolymphomas arise as well as case-specific ancillary studies help to minimize this misinterpretation. Here, we report on an unusual pseudolymphoma in the context of a necrotizing fasciitis of the thigh that was initially misclassified as a lymphoma.

## 2. Case presentation

A 45-year-old male was admitted to the emergency department, presenting with septic shock. Blood values indicated severe inflammation (C-reactive protein 345 mg/L, reference < 5mg/L; leucocytes 1.47 G/L, reference 3–10.5 G/L) and tissue damage (lactate dehydrogenase 676 U/L, reference < 250 U/L; Creatinine kinase 19’055 U/L, reference < 190 U/L; creatinine 349 mcmol/L, reference 59–104 mcmol/L). A computed tomography scan showed signs of infection in the right major psoas muscle and the muscles of the proximal thigh along with an enlarged inguinal ipsilateral lymph node. No other suspicious lymph nodes were noted in complementary imaging. No PET-CT scan was performed. Surgical debridement of the necrotic tissue and excision of the enlarged lymph node was performed. At the intensive care unit antibiotic treatment for necrotizing fasciitis and hemofiltration was carried out and the wound was repeatedly debrided. The patient’s general condition improved and he was finally discharged into rehabilitation. The patient’s past medical history included metastatic testicular mixed germ cell tumor treated with chemotherapy two years ago. The prior routine follow-up several weeks before the current presentation had been unremarkable. No palpable lymphadenopathy or hepatosplenomegaly was noted and the patient reported no B - symptoms. The last follow up visit approximately one year after the incident was unremarkable.

**Figure 1.**
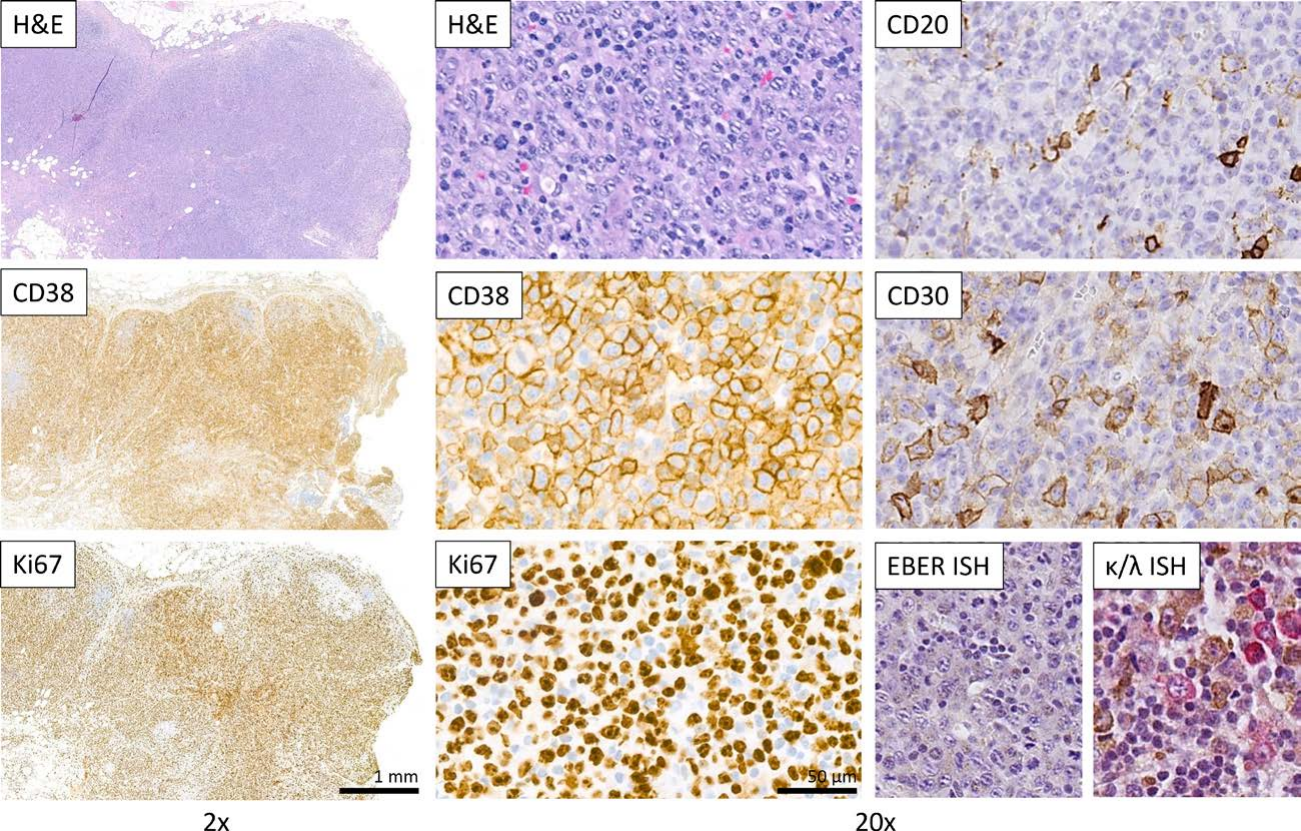
Hematoxylin and eosin (H&E) staining and immunohistochemical analysis of the excised lymph node. Left panel: low-power view of H&E, CD38 and Ki67 (proliferation marker; positive reaction shows brown coloration) reveals a proliferation of plasmablast-like cells with effacement of the nodal architecture. Middle panel: high-power view (20× objective) of the plasmablast-like population. Most of the cells negative for CD38 and Ki67 correspond to interfollicular reactive T-cells. Right panel: The plasmablast-like proliferation is negative for CD20 and shows a variable positivity for CD30. The EBER in situ hybridization was negative, the kappa and lambda in situ hybridization highlights both kappa (brown coloration) and lambda (red coloration) expressing plasmablast-like cells.

**Figure 2.**
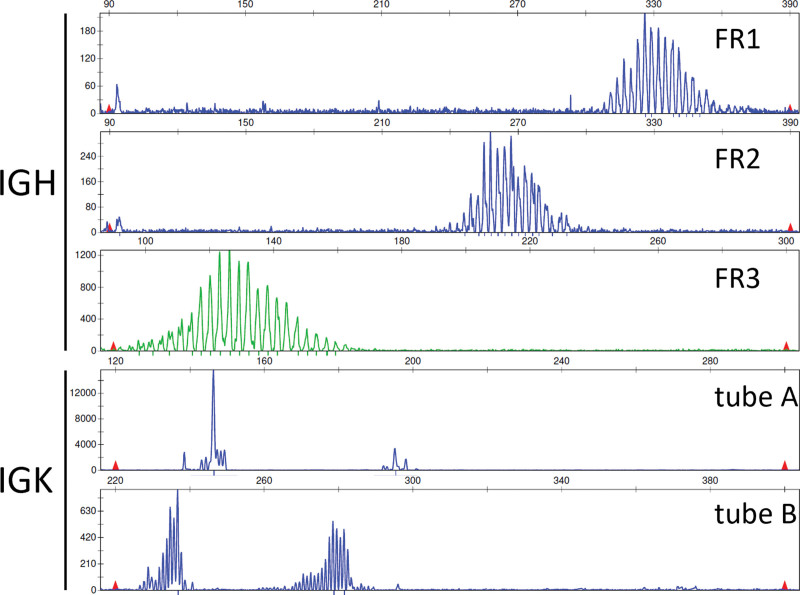
Multiplex PCR for detection of clonal immunoglobulin gene rearrangements according to the BIOMED-2 protocol. Upper 3 panels: Multiplex PCR results for IGH (VH-JH) rearrangements show a polyclonal pattern for IGH VH framework regions (FR) 1-3. Lower 2 panels: Multiplex PCR results for IGK rearrangements show a polyclonal pattern for Vκ-Jκ (tube A) and Vκ/intron-Kde (tube B) rearrangements. The dominant peak at 146bp in tube A does not indicate clonality, as it represents the most common IGK rearrangement and is evident in the polyclonal control as well (data not shown). Multiplex PCR reactions and primers were carried out and designed according to the BIOMED-2 Concerted Action BMH4-CT98-3936 protocol. IGH = immunoglobulin heavy locus, IGK = immunoglobulin kappa locus.

The histologic findings in the resected adipose and muscular soft tissue were compatible with necrotizing fasciitis. The macroscopic evaluation of the lymph node specimen revealed one single, enlarged (3.0 cm diameter), ovaloid lymph node with a whitish homogenous cut surface. Histologic sections demonstrated a largely effaced lymph node architecture with subcortical nodular aggregates of Langerhans cells and some residual B-cell areas with retained meshworks of follicular dendritic cells. Most of the lymph node was infiltrated by sheets of blastoid and plasmablast-like cells, respectively, with immunohistochemical expression of CD79a, CD38, VS38, MUM-1 and a proliferation index (Ki67) of ≥ 95% (Fig. 1). The plasmablast-like cells were variable positive for CD30, weakly and only partially positive for PAX5, BOB1 and OCT2, and negative for CD20, c-MYC, CyclinD1, EMA, ALK, CD56, all investigated T-cell markers (CD2, CD3, CD4, CD5, CD8) and T-follicular helper cell markers (CD57, PD1). Epstein-Barr encoding region in situ hybridization was negative. The morphological worrisome features with architectural effacement and the very high proliferation rate were interpreted as signs of malignancy and the case was signed out as “malignant lymphoma; further workup required for final classification.” Additional analyses were carried out and the slides were reviewed by another board-certified pathologist. Immunohistochemical and in situ analysis of kappa and lambda immunoglobulin light chains were compatible with a polyclonal expression pattern. The fluorescence in situ hybridization with break-apart probes for BCL2, BCL6, and MYC did not reveal evidence for a rearrangement of these genes. The BIOMED-2 standardized immunoglobulin gene rearrangement detection system did not show a clonal rearrangement for Immunoglobulin Heavy Locus or Immunoglobulin Kappa Locus (Fig. 2). The results of these studies did not support the initial morphological diagnosis of lymphoma and a pseudolymphoma with a very unusual proliferation of plasmablasts/blastic lymphoid cells in the context of necrotizing fasciitis was suggested. Due to the unusual presentation and the worrisome proliferation rate of the atypical lymphoid cells, the case was sent out for external review. The results of the additional studies performed by a lymphoma reference center (immunohistochemical stainings for the IgD, IgA, IgM, IgG immunoglobulin heavy chains) confirmed a non-clonal blastic population The integrative diagnosis, taking into account morphological, immunohistochemical and molecular findings within the clinical context was that of an unusual reactive lymphadenopathy with a massive activation/polyclonal proliferation of blasts and plasmablasts, respectively. The criteria for the diagnosis of a lymphoma were not met. There was no morphologic or immunohistochemical evidence for a manifestation of the known testicular germ cell tumor. The clinical follow-up six months later was unremarkable, with no evidence of residual disease.

## 3. Discussion

This case report illustrates the pitfalls in the histopathologic interpretation of an enlarged lymph node specimen – in particular in the drainage area of a site of acute infection, in this case in the context of severe necrotizing fasciitis. The worrisome histological findings initially caused a misclassification of a reactive lymphadenopathy as an aggressive lymphoma.

The histopathologic diagnosis of a lymphoma and its accurate classification remains challenging for a substantial percentage of cases. However, precise classification is essential for the optimized and specific clinical management of lymphoma patients.^[1,2]^ The distinction between reactive and neoplastic lymphoproliferations can be difficult by clinical and histological means. The clinical and radiologic findings of various benign conditions may mimic malignancy, including but not limited to autoimmune diseases, benign lymphoproliferative disorders, infectious diseases and drug hypersensitivity reactions.^[3,4]^ Classic histologic mimics of lymphoma include the polymorphous lymphatic proliferations in the context of acute EBV infection, reactive follicular and interfollicular hyperplasia, the proliferative phase of a Kikuchi histiocytic necrotizing lymphadenitis, IgG4-related lymphadenopathy and the rare autoimmune lymphoproliferative syndrome. These differential diagnoses have been extensively reviewed elsewhere.^[4–8]^ Besides these known mimics, our case reports illustrates that also nonspecific acute bacterial infections can stimulate a polyclonal expansion of blastoid B-cells in the affected draining lymph node to such an extent, that a lymphoma is a genuine differential diagnosis. This specific immunoreactive pattern with a lymph-node effacing activation of blasts and polyclonal proliferation of short-lived plasmablasts has not specifically been discussed in the literature as a mimic of lymphoma. The main differential diagnosis in our case included “aggressive” CD20-negative lymphomas with a plasmablastic morphology, namely ALK-positive large B-cell lymphoma and plasmablastic lymphoma, as well as a primary CD20-negative diffuse large B-cell lymphoma.^[9,10]^ The first two lymphoma entities were unlikely as the plasmablastic proliferation was negative for ALK, EMA and Epstein-Barr encoding region in situ hybridization. The presence of subcortical nodular aggregates of Langerhans cells, the polytypic expression and lack of a clonal rearrangement of the of Immunoglobulin light and heavy chains pointed in favor of a reactive process, and argued against a diagnosis of a primary CD20-negative diffuse large B-cell lymphoma or an unclassifiable lymphoma. After reviewing the clinical information in the interdisciplinary tumor board, placing the findings in the accurate context and with the help of additional immunohistochemical and molecular analyses the final diagnosis of a reactive lymphadenopathy could be made.

## 4. Conclusion

This unusual case underlines the difficulties in diagnosing lymphomas and supports the importance of interdisciplinary teamwork. It adds the parafollicular and lymph node architecture-effacing activation of blasts and polyclonal proliferation of short-lived plasmablasts during an acute bacterial infection to the potential mimics of lymphomas.

## Acknowledgments

This case report was presented at the Swiss Academy of multidisciplinary Oncology (SAMO) Workshop on Lymphoma 2021 in Lucerne, Switzerland. The authors thank the workshop organizers for the event and lively discussion of the presented case.

## Author contributions

All authors contributed to the conception, design and critical review of the manuscript. All authors read and approved the final version of the manuscript.

**Conceptualization:** Bastian Dislich, Yara Banz.

**Investigation:** Bastian Dislich, Dennis Hoch, Stefan Dirnhofer, Urban Novak, Yara Banz.

**Methodology:** Bastian Dislich, Dennis Hoch, Stefan Dirnhofer, Yara Banz.

**Resources:** Stefan Dirnhofer.

**Supervision:** Bastian Dislich, Stefan Dirnhofer.

**Visualization:** Bastian Dislich.

**Writing – original draft:** Bastian Dislich.

**Writing – review & editing:** Bastian Dislich, Dennis Hoch, Stefan Dirnhofer, Urban Novak, Yara Banz.
